# Genetic diversity and evolution of the virulence plasmids encoding aerobactin and salmochelin in *Klebsiella pneumoniae*

**DOI:** 10.1080/21505594.2021.1924019

**Published:** 2021-05-10

**Authors:** Dongxing Tian, Meng Wang, Ying Zhou, Dakang Hu, Hong-Yu Ou, Xiaofei Jiang

**Affiliations:** a Department of Laboratory Medicine, Huashan Hospital, Shanghai Medical College, Fudan University, Shanghai, China;; b State Key Laboratory of Microbial Metabolism, Joint International Laboratory on Metabolic & Developmental Sciences, School of Life Sciences & Biotechnology, Shanghai Jiao Tong University, Shanghai, China

**Keywords:** Hypervirulent *Klebsiella pneumoniae*, virulence plasmid, genome evolution, epidemiology, conjugative transfer

## Abstract

Virulence plasmids of hypervirulent *Klebsiella pneumoniae* (hvKp) have the potential to transfer to drug-resistant strains or integrate with other plasmids, facilitating the genome evolution of threatening pathogens. We conducted an in-depth analysis of the publicly available 156 complete genome sequences of hvKp together with a multi-region clinical cohort of 171 hvKp strains from China to provide evidence for the virulence plasmid evolution. Virulence plasmids were frequently detected in the ST23 and ST11 *K. pneumoniae* strains. Multidrug-resistant hvKp (MDR-hvKp) occupied a large proportion of hvKp, and the coexistence of virulence and resistance plasmids may be the major cause. Virulence plasmids commonly possessed multiple replicons, of which IncFIB_K_ was the most prevalent (84.6%). We identified 49 IncFIB_K_ alleles among 583 IncFIB_K_ plasmids, and they could be divided into Clades I, II, and III. We further observed that conjugative and non-conjugative virulence plasmids could be distinguished by IncFIB_K_ genetic diversity, and IncFIB_K_ subtyping could also indirectly indicate a chimeric preference of conjugative virulence plasmids. On this basis, we developed an open-access web tool called *KpVR* for IncFIB_K_ subtyping. In conclusion, the genetic diversity of IncFIB_K_ virulence plasmids could be used for tracking the evolution of virulence plasmids, and further preventing the emergence of MDR-hvKp strains.

## Introduction

Two major pathotypes of *Klebsiella pneumoniae* pose serious hazards to public health: hypervirulent *K. pneumoniae* (hvKp) causes severe invasive infections while classic *K. pneumoniae* (cKp) are mostly hospital-acquired and multidrug-resistant (MDR) [1]. The mobilizable plasmids are the widely acknowledged cause of the dissemination of the resistance genes among cKp [2,3]. Multiple factors are required for hypervirulence of hvKp, such as iron acquisition and capsular polysaccharide [4]. The critical virulence factors with experimental support for conferring the hypervirulent phenotype are encoded by genes present on the virulence plasmids, which include *iuc* (coding for aerobactin), *peg344* (a metabolic transporter), and *rmpA* and *rmpA2* (regulators of capsule production) [5]. Virulence plasmid acquisition may be an important mechanism for the increased virulence of hvKp. Plasmids carrying resistance or virulence genes have the potential to be transferred between bacterial strains, conferring resistance and virulence.

However, the patient survival of infections with strains displaying both hypervirulence and multi-drug resistance is unexpectedly poor. Indeed, Gu et al. has reported that carbapenem-resistant ST11 *K. pneumoniae* strains showed increased virulence after acquiring a pK2044-like plasmid, resulting in extremely difficult treatment and high mortality of such infections [6]. Nevertheless, current thinking considers pK2044-like virulence plasmids without conjugation and mobilization genetic modules to be non-transmissible [7]. Determining how classical pK2044-like virulence plasmids show mobility remains to be further studied. The escalating threat to global health posed by hypervirulent *K. pneumoniae* has warranted urgent investigation and recent research has uncovered some important findings. Clonal Group 23 (CG23) has been described as having a strong association with hvKp strains, causing primary liver abscess [8,9]. Efficient iron acquisition is required for the survival of hvKp in human ascites [10]. Thus, the ability of hvKp to produce more iron acquisition factors could enhance its virulence. Several molecular epidemiologic studies showed that aerobactin and salmochelin siderophore genes *iuc* and *iro* encoded on plasmids are hvKp speciﬁc, and could be useful as clinical biomarkers for hypervirulent *K. pneumoniae* strains [4,11]. Further, Russo et al believed that *iuc* and/or either *rmpA* or *rmpA2* would be predicted to be the best biomarkers. In addition, based on genetic diversity, Lam and Wyres et al. developed a framework for identifying and tracking key virulence loci encoding aerobactin and salmochelin [12]. The authors further noted that the most common pK2044-like classical virulence plasmid (KPVP-1) almost always harbored an IncFIB_K_ replicon. In fact, IncFIB_K_ plasmid is widespread in *Klebsiella* strains, and most of the IncFIB_K_ plasmids are found to carry resistance genes [13,14]. The genetic diversity of IncFIB_K_ replicons could help us understand the differences between the IncFIB_K_ virulence plasmids and the IncFIB_K_ resistance plasmids. Other types of virulence plasmids were also reported, such as IncFIB_Mar_ [15]. However, comprehensive analysis of virulence plasmids is still lacking.

The virulence plasmid acquired by ST11 carbapenem-resistant *K. pneumoniae* was a classical non-conjugative pK2044-like virulence plasmid. Besides, self-transmissible virulence plasmids have also appeared. For example, Yang et al. identified that a conjugative virulence plasmid from *K. variicola* strain could be transferred to carbapenem-resistant *K. pneumoniae* [16], indicating the rapid evolution of virulence plasmids. Therefore, it has become increasingly urgent to understand the genetic diversity of conjugative and non-conjugative virulence plasmids and further to quickly identify conjugative virulence plasmids in clinical isolates.

In this study, we observe that virulence plasmids are found in a wide range of host bacteria, not limited to CG23 strains, and MDR-hvKp has occupied a considerable proportion of hvKp. IncFIB_K_ replicon is dominant in virulence plasmids, and the genetic diversity of their replicons could distinguish conjugative from non-conjugative virulence plasmids. We also described a new open-access online tool *KpVR* for IncFIB_K_ subtyping. Our results might significantly deepen our understanding of the evolution of virulence plasmids of *K. pneumoniae* and provide insights for further research into hypervirulent *K. pneumoniae.*

## Methods

### GenBank data and clinical isolates

Completely sequenced virulence plasmids with lengths more than 50 kb were extracted from GenBank by searching *iuc, iro, rmpA, and rmpA2* in July 2020 (https://www.ncbi.nlm.nih.gov/nuccore). Complete chromosome sequences were obtained for some of them (Datasets S1 and S2). We focused on the two most common specific virulence determinants for iron acquisition, *iuc* and *iro*, encoded on the plasmids of hvKp. Other virulence genes were not analyzed in this study.

According to previous studies, we used *iuc* and/or *iro, rmpA*, and *rmpA2* to screen for clinical hvKp strains [4]. *K. pneumoniae* clinical isolates were collected in nine hospitals from seven Chinese provinces from January 2017 to February 2018. A total of 530 isolates were collected from Huashan Hospital (Shanghai), Sixth Hospital of Shanxi Medical University (Shanxi), Taizhou Municipal Hospital (Zhejiang), Taizhou Municipal Hospital (Zhejiang), Kunming Yan’an Hospital (Yunnan), First Affiliated Hospital of Xiamen University (Fujian), Jinshan Hospital (Shanghai), Shandong Provincial Hospital (Shandong), First Afﬁliated Hospital of Guangxi Medical University (Guangxi) (271, 60, 52, 43, 31, 29, 27, 27, and 19 isolates from each location, respectively).

### Core single nucleotide polymorphisms (SNPs) extraction and phylogenetic analysis

Chromosomal MLST was performed in the Institute Pasteur MLST database online (https://bigsdb.pasteur.fr/klebsiella/). To obtain a further evolutionary relationship among the closely related ST11 strains, genomes of them were used to construct phylogenetic tree based on the genome-wide SNPs. SNPs situated in the core genome region were automatically called and aligned by submitting ST11 genomes to the ‘CSI Phylogeny 1.4ʹ web-based program with disabled prune selection, using the NJST258_1 complete genome as a reference [17]. The alignments were released from CSI Phylogeny and then used to construct maximum-likelihood trees with 1000 bootstrap values using MEGA v7.0.26 software [18].

### Identification of plasmid replicons and iuc and iro alleles

Virulence plasmid replicons were identified using the PlasmidFinder database using the minimum coverage and minimum identities of 90% (https://cge.cbs.dtu.dk/services/PlasmidFinder/) [19]. Acquired antibiotic resistance genes were identified using CRAD 2020 with the default threshold [20]. MDR was defined as resistant to three or more antimicrobial classes [21]. Virulence genes (*iro* and *iuc*) were typed using *Kleborate* v0.3.0 (https://github.com/katholt/Kleborate/) [12].

### Phylogenetic analysis of virulence plasmids

Twenty-five plasmids were selected according to the replicons and MLSTs of the host bacteria. The phylogenetic tree was constructed according to the replicon sequences (IncFIB and IncFIA) of the reference plasmids using the maximum-likelihood method with 1000 bootstrap replicates in MEGA7. Visualization, annotation, and management of the phylogenetic tree were performed by Evolview v3 (https://www.evolgenius.info/evolview).

### Subtyping IncFIB_K_ plasmids in Klebsiella bacteria

To obtain a comprehensive overview of IncFIB_K_ plasmids, the complete sequences of IncFIB_K_ plasmids of *Klebsiella* from GenBank were selected by BLASTn using the reference replicon “IncFIB_K__1_JN233704” in the PlasmidFinder database (≥90% identities and ≥90% coverage) (Dataset S4). IncFIB_K_ alleles were determined by the nucleotide sequence identity using “IncFIB_K__1_JN233704” as a reference, and any replicon sequence with single nucleotide variations or deletions after multiple-sequence alignment by ClustalW was defined as a new allele (Figure S1). We developed a user-friendly web-based tool, named *KpVR* (https://db-mml.sjtu.edu.cn/KpVR/), as a public resource for detecting replicons, T4SS gene clusters, virulence, and antibiotic resistance genes in *K. pneumoniae* plasmids. First, we developed a back-end data set repDB based on our IncFIB_K_ subtypes. Next, the online tool *KpVR* performs rapid similarity searches of a query plasmid sequence against repDB based on nucleotide sequence identity and coverage. Then, *iuc* and *iro* lineages based on the AbST and SmST typing schemes were also integrated by *KpVR* [12]. Finally, *KpVR* outputs a simple list and generates a graphic overview of the prediction of replicon types together with the extended putative virulence modules or acquired antibiotic resistance genes. All IncFIB_K_ virulence plasmids in this study were submitted into *KpVR* to acquire corresponding alleles.

IncFIB_K_ subtyping primers for clinical isolates were designed according to the conserved regions at least 50bp away from both sides of the “IncFIB_K__1_JN233704” sequences. The sequences of preselected primer regions of IncFIB_K_ plasmids were extracted and then visualized using WebLogo to determine the primer sequences (Figure S2). Forward primer: GCCTTRATGACTTCGTCATA; reverse primer: CRGACGTTAAGATCACCGG. The positive PCR products were sequenced and then submitted to *KpVR* to determine the IncFIB_K_ alleles.

### Comparative analysis of conjugative virulence plasmid genomes

Four conjugal modules in the plasmid sequences were detected by using oriTfinder [22], including the origin of transfer site (oriT), relaxase gene, type IV coupling protein (T4CP) gene, and the type IV secretion system gene cluster (T4SS). BLAST Ring Image Generator (BRIG) was used to compare conjugative virulence plasmids with other similar plasmids to further generate circular plasmid maps.

## Results

### General characteristics of hypervirulent strains and virulence plasmids of K. pneumoniae

**Figure 1.**
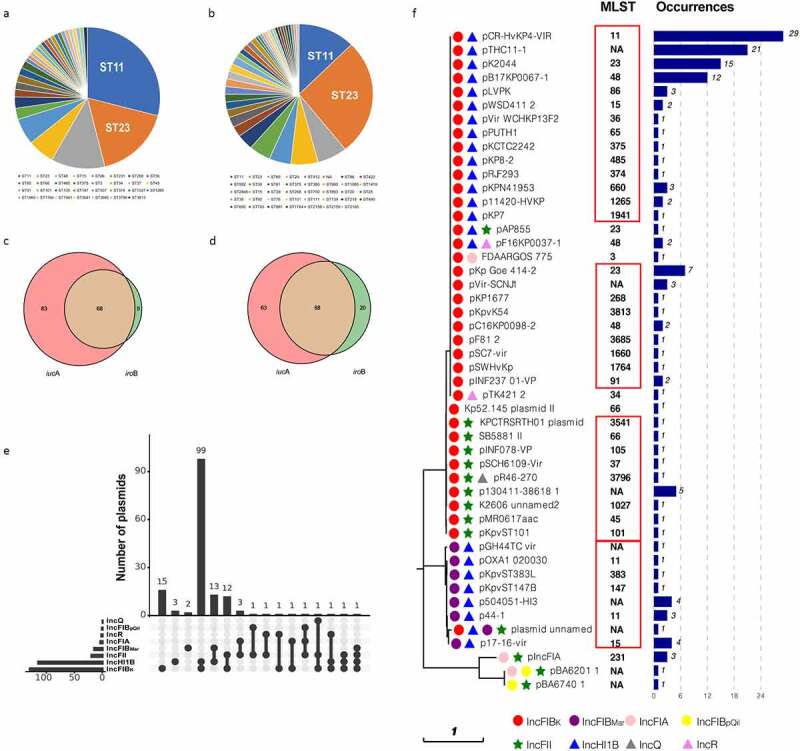
**The distribution of hvKp strains and virulence plasmids**. MLST of the 119 completely sequenced hvKp strains available in GenBank (a) and 171 clinical hvKp isolates (b). The distribution of *iuc*A and *iro*B in GenBank (c) and clinical strains (d). (e) The replicons of the virulence plasmids. The dot matrix shows different multi-replicon profiles and the histograms on the top show the numbers of plasmids with the corresponding profiles. Histograms on the left represent numbers of corresponding replicons. (f) The occurrences of distinct virulence plasmids in various ST clones. The tree was constructed by using the maximum-likelihood method with 1,000 bootstrap replicates in MEGA7. The replicons are represented by different shapes and colors. The same type of virulence plasmids is framed in red

We identified 156 fully sequenced virulence plasmids with various lengths (70 to 479 kb; median, 213 kb) and GC contents (46.4% to 53.1%; median, 49.9%)(Figure S3), and 117 complete chromosomes of them were then extracted for analysis (Datasets S1 and S2, Figure S1). The strains were isolated from f14countries and regions between 2006 and 2019 (Figure S4A). More than 30 ST types were identified from 117 *K. pneumoniae* chromosomes, among which ST11 and ST23 accounted for 29.9% and 16.2%, respectively (Figure 1A). Given the high proportion and concentrated distribution of ST11 strains, we conducted geographical distribution and core SNPs phylogenetic tree analyses of these strains to exclude the possibility of clonal dissemination (Fig S4B and Figure S4C).

Besides the GenBank data, we also identified 171 hvKp strains from 573 non-duplicated *K. pneumoniae* clinical isolates to further investigate ST distribution trends. The overall distribution of ST types detected in clinical hvKp isolates was also similar to the global data (Figure 1B). ST11 still accounted for a large proportion of clinical hvKp isolates (12.3%; 21/171) being second only to ST23 (26.3%; 45/171).

We noticed that most *iuc*/*iro* positive virulence plasmids also carry *rmp*A or *rmp*A2, except those plasmids carrying *iuc*3/*iuc*5 loci (Dataset S2). The different distribution of *iuc* and *iro* was also observed. There were more hvKp strains carrying *iuc*A (96.2%; 151/156) than those carrying *iro*B (46.8%; 73/156) (GenBank) (Figure 1C). Highly similar findings were observed in clinical hvKp strains (Figure 1D). Strains carrying both *iuc*A and *iro*B accounted for 43.6% (68/156) and 51.5% (88/171) in GenBank and clinical isolates, respectively.

### The emergence of MDR-hvKp strains

The dual phenotype of virulence and resistance may occur when the virulence plasmids carry resistance genes or exist in the same strain with the MDR plasmids. We observed that 90 of 117 hvKp strains were identified as MDR-hvKp, and 23 of them had resistance genes in their virulence plasmids (Figure S5, Dataset S3). The coexistence of virulence and resistance plasmids in the same strain may be the major cause of MDR-hvKp.

### The predominance of IncFIB_K_ virulence plasmids

Virulence plasmids commonly possess multiple replicons, and recognizable replicon types included IncFIB_K_, IncFIB_Mar_, IncFIB_pQil_, IncHI1B, IncFIA, IncFII_K_, IncFII_pKP91_, IncFII_pAMA1167-NDM-5_, IncFII_pHN7A8_, IncR, and IncQ. For simplicity, IncFII_K_, IncFII_pKP91_, IncFIIp_AMA1167-NDM-5_, and IncFII_pHN7A8_ were hereafter grouped into IncFII. The two most prevalent replicons detected were IncFIB_K_ (84.6%; 132/156) and IncHI1B (75%; 117/156). Notably, they were always found together, making IncFIB_K_/IncHI1B plasmids the most prevalent virulence plasmids (63.5%; 99/156) (Figure 1E). Together with other replicons, the plasmids could be classified into s16types (Figure 1E).

We further observed that the same plasmid type could be found in various ST *K. pneumoniae* strains. For example, pK2044-like IncFIB_K_/IncHI1B virulence plasmids were not restricted to ST23 but also occurred in ST11, ST48, and other strains, indicating that the virulence plasmids may have been transferred (Figure 1F). Another important finding was that the IncFIB replicon (95.5%; 149/156), including IncFIB_K_, IncFIB_Mar_, and IncFIB_pQil_, was nearly always present in each virulence plasmid (Figure 1F), highlighting that the IncFIB replicon likely plays a crucial role in the formation and replication of the virulence plasmid.

### The replicons of IncFIB_K_ virulence plasmids

Considering the widespread distribution of IncFIB_K_ replicon in *Klebsiella*, we collected 583 fully sequenced IncFIB_K_ plasmids restricted to *Klebsiella* from the GenBank database to explore the genetic divergence between virulence and non-virulence IncFIB_K_ plasmids (Dataset S4). We identified 49 IncFIB_K_ alleles by nucleotide identity, and single nucleotide variations accounted for most of the variations in these alleles (Figure S1).

**Figure 2.**
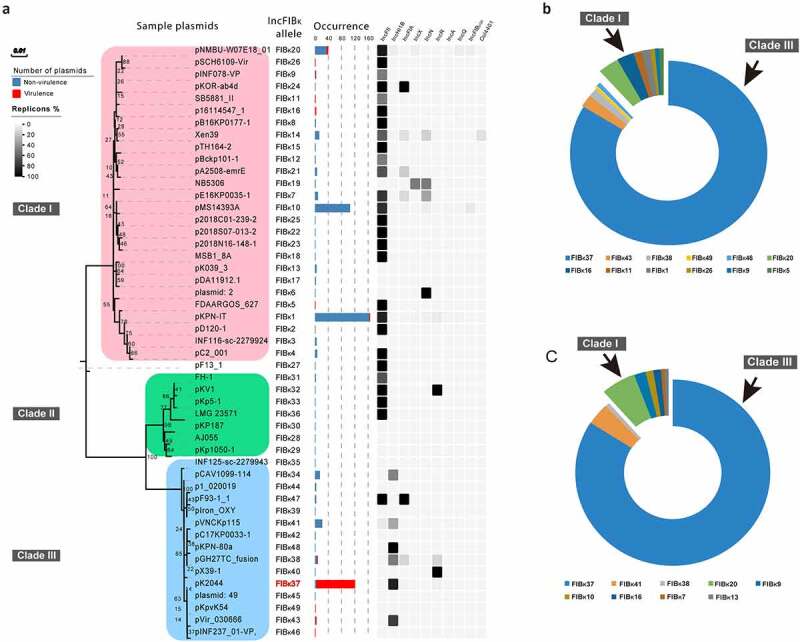
**Descriptions of IncFIB_K_ alleles**. (a) Phylogenetic tree, occurrences, and other replicons of IncFIB_K_ plasmids. On the left is the phylogenetic tree of all IncFIB_K_ alleles. The tree was constructed by using the maximum-likelihood method with 1,000 bootstrap replicates in MEGA7. Colors indicate Clade I, II, and III from up to bottom. The next bar plots are the occurrences of the corresponding plasmids. Colors indicate virulence and non-virulence plasmids. Other co-occurring replicons were shown on the right. (b) IncFIB_K_ alleles in IncFIB_K_ virulence plasmids available in GenBank. (c) IncFIB_K_ alleles of virulence plasmids in clinical hvKP isolates

Phylogenetic tree analysis showed that the 49 IncFIB_K_ replicon alleles were grouped into three clades (Clade I, Clade II, and Clade III) (Figure 2A). Of these, IncFIB_K_1, IncFIB_K_10, and IncFIB_K_37 were the three most common. A total of 12 IncFIB_K_ alleles were identified in 132 IncFIB_K_ virulence plasmids by *KpVR*. Unexpectedly, nearly all virulence plasmids were found with IncFIB_K_37, a branch in Clade III, while the general non-virulence IncFIB_K_ plasmids belonged to Clade I (Figure 2A, Figure 2B). Plasmids in Clade I often carried IncFII replicons simultaneously (Figure 2A).

To verify the observations in the GenBank data that IncFIB_K_ replicons in Clade III, especially IncFIB_K_37, were highly associated with virulence plasmids, we identified IncFIB_K_ alleles in 171 hvKp clinical isolates using the web tool *KpVR*. Indeed, 91.8% (157/171) of clinical hvKp isolates were found to harbor IncFIB_K_ replicons, and 11 IncFIB_K_ alleles were identified in IncFIB_K_-positive clinical hvKp isolates. Of these, the IncFIB_K_37 allele accounted for the highest proportion (84.1%; 132/157) (Figure 2C). It is important to note that a small number of IncFIB_K_ virulence plasmids belonging to Clade I were found in both GenBank data and clinical isolates (Figure 2B Figure 2C).

### Genetic diversity of conjugative and non-conjugative IncFIB_K_ virulence plasmids

**Figure 3.**
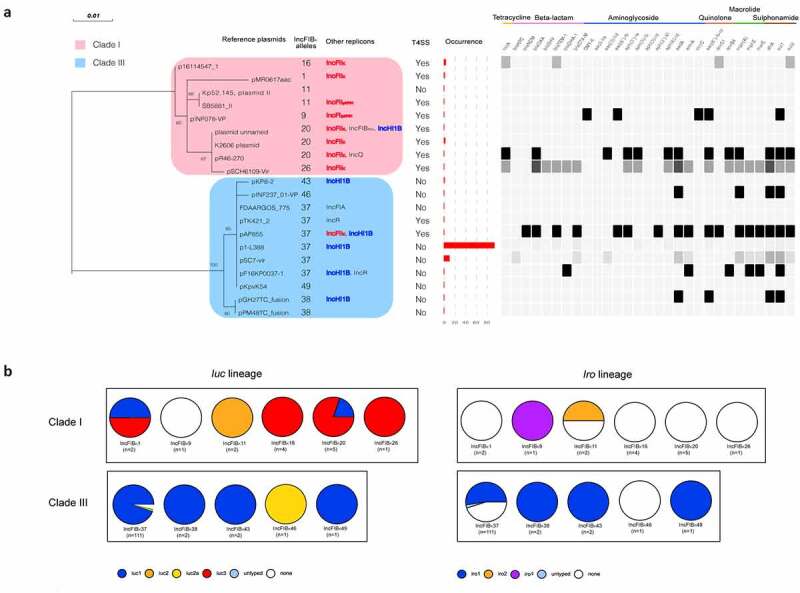
**IncFIB_K_ virulence plasmids in distinct genetic groups**. (a) The differences of AMR genes and other replicons of IncFIB_K_ virulence plasmids in Clade I and Clade III. (b) The *iuc* and *iro* genes of IncFIB_K_ virulence plasmids in Clade I and Clade III. Different colors indicate different lineages of *iuc* and *iro* genes

Virulence plasmids in Clade I generally carried IncFII replicons while Clade III carried IncHI1B replicons, and all IncFIB_K_/IncFII virulence plasmids in Clade I had the transfer region (Figure 3A). Therefore, almost all Clade I virulence plasmids were conjugative plasmids while virulence plasmids in Clade III were non-conjugative. In addition, we identified 27 conjugative virulence plasmids, all but one of which were concentrated in Clade I, providing indirect evidence for the significant genetic diversity between conjugative and non-conjugative virulence plasmids (Table S1). Collectively, these findings indicate that classical non-conjugative virulence plasmids and conjugative virulence plasmids belonged to Clade III and Clade I, respectively. The IncFIB_K_ appears to a large extent to discriminate between conjugative and non-conjugative virulence plasmids. Some conjugative virulence plasmids seemed to carry some resistance genes (Figure 3A).

The loci and distribution of *iuc* and *iro* of IncFIB_K_ virulence plasmids in Clade I and Clade III also showed divergences. Plasmids in Clade I almost harbored *iuc* genes, but few of them carried *iro* genes. In contrast, plasmids in Clade III tended to carry both *iuc* and *iro* genes. Besides, *iuc* lineages of plasmids in Clade I consisted largely of *iuc*3 while those in Clade III were mainly *iuc*1 (Figure 3B).

### Chimeric preference of conjugative IncFIB_K_ virulence plasmids

We observed above that except for plasmid pAP855, all conjugative virulence plasmids were concentrated within Clade I. We compared the conjugative plasmids p205880-1 (Clade I) and pAP855 (Clade III) to other related plasmids to explore the differences between them.

**Figure 4.**
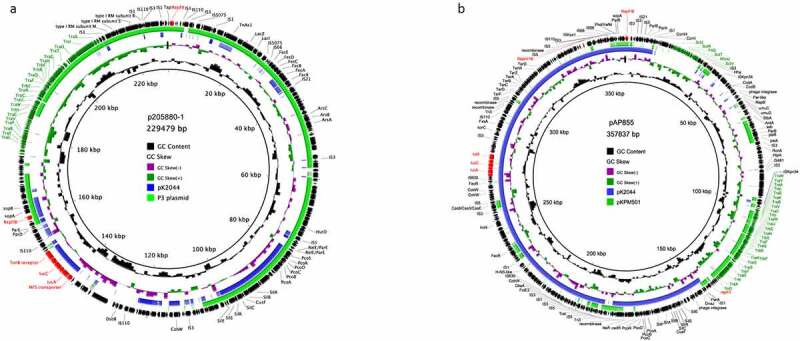
**Comparative analysis of conjugative virulence plasmid with other similar plasmids**. (a) Comparative analysis of conjugative virulence plasmid p205880-1 in Clade I. The plasmid p205880-1 (accession: CP030303.1) was used as the reference plasmid to perform genome alignment with classical virulence plasmid pK2044 (accession: AP006726.1) and IncFII_K_ P3 plasmid (accession: CP059380.1). (b) Comparative analysis of conjugative plasmid pAP855 in Clade III. The plasmid pAP855 (accession: CP035384.1) was used as the reference plasmid to perform genome alignment with classical virulence plasmid pK2044 (accession: AP006726.1) and IncFII_K_ plasmid pKPM501 (accession: CP031735.1)

Plasmid pAP855 (Clade III) was a very large 357.8 Kb co-integrated plasmid which could be possibly disassembled into a backbone of a pK2044-like plasmid (Clade III) and an IncFII *tra-trb* conjugative transfer region (Figure 4B). In contrast, the Clade I conjugative plasmid p205880-1 may have evolved through the acquisition of virulence genes by a Clade I IncFIB_K_/IncFII plasmid (Figure 4A). Further, we found that other conjugative virulence plasmids in Clade I exhibited high similarity with p205880-1. All shared an almost identical aerobactin-related virulence module integrating into a classical non-virulence IncFIB_K_/IncFII plasmid with only one exception involving plasmid pINF078-VP which carried 13 copies of *iro* genes (Figure S6). Together these data indicate that the Clade I conjugative plasmids likely evolve into conjugative virulence plasmids by acquiring an aerobactin-related virulence cluster while non-conjugative virulence plasmid pAP855 acquire a transfer region.

## Discussion

HvKp infections are not only restricted to Asia but occur worldwide [8,23]. Since the report of a fatal outbreak of the carbapenem-resistant hypervirulent *K. pneumoniae* (CR-hvKp) ST11 strain in a Chinese hospital in 2016, MDR-hvKp infections, especially those caused by CR-hvKp, have been identified in many other countries including Singapore [24], Iran [25], France [26], and Russia [27], providing a global wake-up call for the widespread dissemination of MDR-hvKp strains. The infections caused by MDR-hvKp would exacerbate poor clinical outcomes, highlighting the need to understand the role of virulence plasmids in these “dual risk” hypervirulent superbugs.

Total siderophore production has been shown to strongly correlate with in vivo virulence. Aerobactin and salmochelin are high-affinity siderophores that are always encoded on virulence plasmids [9]. Although many factors may contribute to the virulence of hvKp, virulence plasmids encoding aerobactin appear to be less dispensable. Our study focuses on virulence plasmids encoding aerobactin and/or salmochelin, incorporating analysis of multi-center Chinese clinical isolates with global GenBank strains. Tracing the host genomes of these plasmids identified a large proportion of ST11. However, ST11 was reported as the most common type of carbapenem-resistant *K. pneumoniae*. Further phylogenetic analysis and clinical data confirmed that ST11 was indeed increased in hvKp strains, second only to ST23 (Fig S4). One previous study showed that ST11 strains possibly acquired a virulence plasmid and exhibited increased virulence [6]. A striking observation is that the same virulence plasmid could be found in various ST types, indicating the likelihood of plasmid transfer between strains. However, virulence plasmids were previously considered to persist for long periods and the lack of conjugation machinery supports their clonal expansion [28]. The common presence of virulence plasmids in drug-resistant *K. pneumoniae*, therefore, highlights the urgency for countering this important global public health threat. Besides, the combination of MDR and virulence could also be caused by the “mosaic plasmid” carrying both antibiotic resistance and hypervirulence-associated features [29]. On the basis of these findings, we comprehensively dissected the composition of different virulence plasmids to look for possible evolutionary clues that could be used to prevent their dissemination.

In this study, IncFIB_K_ virulence plasmids were found to be predominant. According to previous studies, IncFIB_K_ replicons are mostly associated with MDR plasmids in *K. pneumoniae* [30,31]. A recent study found that plasmids encoding *iuc* or *iro* genes usually harbor an IncFIB_K_ replicon [12]. Our study now provides valuable evidence for the important role of the IncFIB_K_ replicon in the formation and replication of virulence plasmids. We used the online tool *KpVR* to determine the genetic diversity of virulence and non-virulence IncFIB_K_ plasmids together with additional information such as acquired resistance genes and Type IV secretion system (T4SS) gene clusters. The Center for Genomic Epidemiology (CGE) databases could also analyze replicons, virulence and antibiotic resistance genes by three separated tools. Besides this information, the tool KpVR could also predict T4SS gene clusters and *iuc* and *iro* subtyping. More importantly, KpVR integrates these results together with IncFIB_K_ subtyping to explore the close connection between conjugative virulence plasmids and IncFIB_K_ genetic diversity. It may further help predict the mobility of virulence plasmids in clinical hvKp strains. IncFIB_K_ replicon sequences were divided amongst three clades according to the phylogenetic tree but virulence plasmids were mostly located to IncFIB_K_37 (Clade III), a different evolutionary branch from those common IncFIB_K_ plasmids, indicating that IncFIB_K_ virulence plasmids were evolved independently. The different distribution of *iuc* and *iro* supported the different evolution of virulence plasmids in Clade I and Clade III. Though IncFIB_K_ replicon always coexists with IncHI1B, IncFIB_K_ possibly fulfills the crucial role in supporting the formation and replication of virulence plasmids rather than IncHI1B. A previous study provided supporting evidence that repHI1B lacks a partitioning system and other compatible replicons are required for replication [32]. Overall, these data provide important clues that help understand the dissemination mechanisms of the hypervirulent and drug-resistant *K. pneumoniae* as well as demonstrating the utility of the *KpVR* tool.

Our results confirm that the emergence of MDR-hvKp is largely caused by the coexistence of the virulence plasmid and resistance plamid in the same strain, and the presence of “mosaic plasmids” occupies a small proportion [33]. However, we could not ignore the terrible consequences of virulence plasmids or “mosaic plasmids” acquiring the ability to transfer. IncFII plasmids are highly likely to harbor transfer regions gain the ability to undertake conjugative transfer [34]. We found all IncFIB_K_/IncFII virulence plasmids were conjugative and therefore had the potential to promote the emergence of MDR-hvKp. Conjugative virulence plasmids have also been reported in other species such as *Escherichia coli* and *Salmonella enterica* [35,36]. Indeed, virulence factors encoding aerobactin, salmochelin in *K. pneumoniae* shared high homology with those that occur in chromosomes or plasmids of other species [15,35,36]. It seems reasonable to assume that these virulence factors could be transferred via mobile genetic elements, such as conjugative plasmids, to evolve into novel hypervirulent strains. One recent study demonstrated that a virulence plasmid with a type IV secretion system in *K. variicola* could be conjugated to a carbapenem-resistant *K. pneumoniae and E. coli* C600 [16]. Therefore, it is crucial to distinguish between conjugative and non-conjugative virulence plasmids. Toward this, we established that IncFIB_K_ subtyping could serve as an effective approach to recognize most conjugative virulence plasmids. This approach will greatly reduce the cost of *de novo* sequencing of whole genomes and enable quick identification of emerging threats to public health.

Conjugative virulence plasmids are thought to possess the two enabling characteristics of conjugative machinery and virulence-related genes. Conjugative plasmids and virulence plasmids, respectively, each possess one of these properties. We found that almost all conjugative virulence plasmids were formed by the insertion of an aerobactin cluster into an IncFIB_K_/IncFII conjugative plasmid (Clade I). The pAP855 (Clade III) was possibly formed by a *tra-trb* conjugative element integrating into a classical pK2044-like virulence plasmid. Thus, IncFIB_K_ could indirectly reveal the chimeric preference of conjugative virulence plasmids. Yang et al. have speculated that homologous recombination events possibly occurred when integrating two plasmid regions into a mosaic plasmid [37]. We did find some homologous regions in several conjugative virulence plasmids, but the exact mosaic mechanism is still waiting to be confirmed. This study has some other limitations. Rather for this study, we focused on the dominant IncFIB_K_ virulence plasmids to track the evolution of conjugative and non-conjugative virulence plasmids by IncFIB_K_ subtyping, while other virulence plasmids were not analyzed due to the small number. The potential bias of GenBank data was unavoidable though we supplemented multi-center isolates to support. More genome sequences are needed to validate our findings.

In conclusion, we observe that ST11 hvKp occupies a high proportion of hvKp strains, second to ST23, and the co-existence of virulence and resistance plasmids is the major cause of the emergence of MDR-hvKp. IncFIB_K_ virulence plasmids are most detected and exhibit a peculiar evolution from other IncFIB_K_ plasmids. We further present the development of a Web tool *KpVR* for IncFIB_K_ subtyping to distinguish conjugative virulence plasmids from classical non-conjugative pK2044-like virulence plasmids. This work might provide novel insights into the evolution of virulence plasmids and will bolster efforts to understand and limit the emergence of infections caused by MDR-hvKp strains.

## Data Availability

The authors confirm that the data supporting the findings of this study are available within the article and its supplementary materials (https://doi.org/10.6084/m9.figshare.14176469.v2).
